# Does Schoolmate and Teacher Support Buffer against the Effect of Adverse Childhood Experiences on Emotional and Behavioural Problems?

**DOI:** 10.3390/ijerph182413009

**Published:** 2021-12-09

**Authors:** Miriama Lackova Rebicova, Zuzana Dankulincova Veselska, Daniela Husarova, Andrea Madarasova Geckova, Danielle E. M. C. Jansen, Jitse P. van Dijk, Sijmen A. Reijneveld

**Affiliations:** 1 Department of Health Psychology and Research Methodology, Medical Faculty, PJ Safarik University, Trieda SNP 1, 040 11 Kosice, Slovakia; zuzana.veselska@upjs.sk (Z.D.V.); daniela.husarova@upjs.sk (D.H.); andrea.geckova@upjs.sk (A.M.G.); 2 Graduate School Kosice Institute for Society and Health, PJ Safarik University, Trieda SNP 1, 040 11 Kosice, Slovakia; j.p.van.dijk@umcg.nl; 3 Olomouc University Social Health Institute, Palacky University in Olomouc, Univerzitni 22, 771 11 Olomouc, Czech Republic; 4 Department of Community & Occupational Health, University Medical Center Groningen, University of Groningen, Antonius Deusinglaan 1, 9713 AV Groningen, The Netherlands; d.e.m.c.jansen@umcg.nl (D.E.M.C.J.); s.a.reijneveld@umcg.nl (S.A.R.)

**Keywords:** schoolmate and teacher support, adverse childhood experiences, adolescence, emotional and behavioural problems

## Abstract

This study aims to explore the associations of schoolmate and teacher support with emotional and behavioural problems (EBP) and whether schoolmate and teacher support affects the associations of adverse childhood experiences (ACE) and of EBP in adolescence. We obtained data from 5220 students aged from 11 to 15 (48.7% boys), who participated in the Health Behaviour in a School-aged Children study (2018, Slovakia). Using logistic regression adjusted for gender, age and family affluence we assessed the modification of the relations of ACE and EBP by schoolmate and teacher support. Schoolmate and teacher support decreased the probability of EBP (Odds Ratios, 95% confidence intervals: 0.76, 0.74|0.79; and 0.86, 0.83|0.89, respectively). However, we found no statistically significant interactions of schoolmate and teacher support regarding the association of ACE with EBP. Schoolmate and teacher support decreased the likelihood of EBP among adolescents but do not buffer the relation of any previous ACE with EBP.

## 1. Introduction

The occurrence multiple adverse childhood experiences (ACEs) can cause a variety of serious emotional and behavioural problems (EBP) during both childhood and adult life [1,2,3,4,5,6]. ACEs include a wide range of negative events that occur at a young age [1]; e.g., abuse and/or neglect of the child, domestic violence towards the youth’s mother, household substance abuse, household mental illness, parental separation/divorce and a household member with a history of jail/imprisonment [1,7]. ACE’s have been shown to have a dose–response relationship with long-lasting effects among adolescents, i.e., increasing levels of exposure (ACEs) are associated with increasing risks of EBP [1,4].

Adolescents spend much of their time at school with their teachers and schoolmates, and the support of both may lead to healthy development [8,9] and better mental health [10,11,12,13]. Many studies have shown that support from teachers and schoolmates, especially a good teacher-student relationship, plays a positive role in the cognitive and social development of children [14,15]. Contact with teachers and schoolmates add to the effects of various interactions with parents and with other peers as a part of a person’s social network [16], all with a potential influence on their mental health. The theory of social support [17] entails that levels of social support from school may have direct effects on health and may also diminish the negative effects on health due to exposure to stressors [18] via its buffering effect [19]. Family-related ACE was especially found to be associated with worse mental health outcomes and the use of psychosocial care [20,21] which is in line with the Tripartite Model of the Impact of the Family on Children’s Emotion Regulation and Adjustment [22]. Difficulties in parental emotional regulation in cases of family-related ACE may be transferred to children by observational learning, may affect the emotional regulation of adolescents and may result in worse mental health. In case of worsened or absent family resources due to ACE, it is important to consider other potential sources of support for adolescents to support their psychosocial development. Several studies also confirmed that the greater the support from teachers and classmates in adolescence, the lower the incidence of emotional [23,24] and behavioural problems [23,25,26]. These findings also align with Bronfenbrenner’s ecological systems theory [16,27,28] that school also has a large effect on how the child grows and develops. Bronfenbrenner’s ecological systems theory entails that adolescents live in a social context in which several factors play an important role, such as the family, teachers, school, peers, community and their support. The available research further suggests that perceived teacher support decreases in older adolescents, whereas schoolmate support seems to remain stable [29,30], and schoolmates provide more support than teachers [8]. Evidence is lacking on whether schoolmate and teacher support can modify the relationship between ACE and EBP and act as a potential buffer.

The aim of this study was therefore to assess the association of schoolmate and teacher support with emotional and behavioural problems, and whether schoolmate and teacher support affects the association of adverse childhood experiences with emotional and behavioural problems among adolescents. We hypothesized that (1) higher schoolmate and teacher support will decrease the likelihood of emotional and behavioural problems and that (2) schoolmate and teacher support will moderate the association between adverse childhood experiences and emotional and behavioural problems.

## 2. Materials and Methods

### 2.1. Sample and Procedure

We used data from the Health Behaviour in School-aged Children (HBSC) study conducted in 2018 in Slovakia. The HBSC used two-step sampling to obtain a representative sample. In the first step, 140 larger and smaller elementary schools located in rural as well as urban areas from all regions of Slovakia were asked to participate. These were randomly selected from a list of all eligible schools in Slovakia, obtained from the Slovak Institute of Information and Prognosis for Education. The school response rate (RR) was 77.9%. In the second step, we obtained data from 8405 adolescents from the fifth to ninth grades of elementary schools in Slovakia in the target group of 11 to 15 years old (mean age 13.43; 50.9% boys). One class from each grade per school was selected. In case of missing responses, the respondents on the variables to be studied were excluded (3185). Of the respondents, 62% filled out all studied variables as analysed in our manuscript. Missing responses may be a result of the respondents deciding to opt-out from filling in certain questions and that a of one hour period being too short to fill out the questionnaire in full. The final sample consisted of 5220 adolescents (mean age 13.02; 48.7% boys). Respondents with missing responses differed only from those without missing responses regarding two control variables—gender and family affluence. The adolescents with missing responses were more often boys and had, on average, lower family affluence.

The study was approved by the Ethics Committee of the Medical Faculty at P.J. Safarik University in Kosice (16N/2017). Parents were informed about the study via the school administration and were able to opt out if they disagreed with their child’s participation. Participation in the study was fully voluntary and anonymous with no explicit incentives provided for participation.

### 2.2. Measures

Emotional and behavioural problems (EBP) were measured with the Strengths and Difficulties Questionnaire (SDQ), which includes 25 items [31], from which we used the 20 difficulty items. Response categories were: not true (0), somewhat true (1), certainly true (2). The resulting score for overall difficulties range from 0 to 40 [32]. The sum score was dichotomized into normal/borderline (0–19) vs. abnormal (20–40). Cronbach’s alpha for the full difficulties scale was 0.73 in our sample.

Adverse childhood experiences were measured by the question: “Have you ever experienced any of the following serious events? (Death of a brother/sister, Death of your father/mother, Death of somebody else you love, Long or serious illness of yourself, Long or serious illness of one of your parents or of someone else close to you, Problems of one of your parents with alcohol or drugs, Repeated serious conflicts or physical fights between your parents, Separation/divorce of your parents, Separation of your parents due work abroad, Moving to another house/flat, or city/village, Transfer to another school) [1,33]. The response categories were “Yes” and “No”. We created a sum score for the number of ACE experienced, with a higher score indicating more ACEs. Consequently, we classified the number of ACEs into three categories: no ACE (0), one or two ACEs (1) and three or more ACEs (2).

Schoolmate support (i.e., social support from classmates) was measured using three statements: “Here are some statements about the students in your class. Please indicate how much you agree or disagree with each one. (The students in my class enjoy being together; Most of the students in my class are kind and helpful; Other students accept me as I am)” [34]. The response categories ranged on a 5-point scale from “Strongly agree” to “Strongly disagree” [35]. A sum score was computed, with a higher score indicating higher schoolmate support. Cronbach’s alpha in our sample was 0.77.

Teacher support (social support from teacher) was measured using three statements: “Here are some statements about your teachers. Please indicate how much you agree or disagree with each one. (I feel that my teachers accept me as I am; I feel that my teachers care about me as a person; I feel a lot of trust in my teachers)” [34]. The response categories ranged on a 5-point scale from “Strongly agree” to “Strongly disagree” [35]. A sum score was computed, with a higher score indicating higher teacher support. Cronbach’s alpha in our sample was 0.83.

The Family Affluence Scale was used as a measure of socioeconomic status. Family affluence was measured using the Family Affluence III (FAS III) [34], which consists of six questions: “Does your family own a car, van or truck?” (Yes/Yes, one/Yes, two or more), “Do you have your own bedroom for yourself?” (Yes/No), “How many computers does your family own?” (None/One/Two/More than two), “How many bathrooms (room with a bath/shower or both) are in your home?” (None/One/Two/More than two), “Does your family have a dishwasher at home?” (Yes/No), “How many times did you and your family travel out of your country for a holiday/vacation last year?” (Not at all/Once/Twice/More than twice). We converted the FAS summary scores to a final score, which has a normal distribution and a range from 0 to 1. We then created tertile categories of low (0 to 0.333), medium (0.334 to 0.666) and high (0.667 to 1) socioeconomic position [36].

### 2.3. Statistical Analyses

First, we described the sample using descriptive statistics. Second, we assessed the associations of the number of ACE and of schoolmate and teacher support with emotional problems and behavioural problems (Model 1). Finally, we explored the change in the associations of ACE with emotional problems and behavioural problems by schoolmate and teacher support separately (Model 2). For the last two steps, we used logistic regression models adjusted for age, gender and family affluence. Statistical analyses were performed using SPSS v.20.

## 3. Results

### 3.1. The Descriptive Characteristics

The background characteristics are shown in Table 1. Table 1 shows that 10.7% of adolescents had an abnormal SDQ score, indicating EBP, while 90.3% of adolescents had a normal/borderline SDQ score. Almost 39% of adolescents had experiences with 3 or more ACEs; 46% of adolescents had experienced at least one ACE, and just over 15% of adolescents had no experienced with ACE.

### 3.2. Associations between the Number of ACEs and of Schoolmate and Teacher Support with EBP

In Model 1, we explored the associations of the number of ACEs and of schoolmate and teacher support with EBP adjusted for gender, age and family affluence. We found significant associations of three or more ACEs with EBP [odds ratios, OR (95%-confidence intervals, CI): 1.92 (1.40|2.63)]. We also found significant associations of schoolmate and teacher support with emotional and behavioural problems separately [0.76 (0.74|0.79); 0.86 (0.83|0.89)]. Schoolmate and teacher support decreased the likelihood of emotional and behavioural problems (Model 1) by 24% and 14%, respectively (Table 2).

### 3.3. Moderation of the Associations between ACE and EBP by Schoolmate and Teacher Support

In Model 2, we assessed whether schoolmate and teacher support influenced the association of ACEs with EBP. We did not find any statistically significant interactions of the number of ACEs and schoolmate and teacher support on EBP (Table 2).

## 4. Discussion

The aim of this study was to explore the association of schoolmate and teacher support separately with EBP and whether schoolmate and teacher support affects the association of ACEs with EBP among adolescents. We found that schoolmate and teacher support separately decreased the probability of EBP; however, did not buffer the association of ACE with EBP.

Our findings corroborate prior studies showing that higher schoolmate and teacher support decreases the probability of EBP in adolescents [23,24,25]. Likewise, children with more friends from school or higher schoolmate support tend to be characterized by fewer depressive symptoms [37,38,39]. Higher teacher support decreases the probability of emotional distress and engagement in deviant and violent behaviours [40,41,42]. Similarly, previous studies revealed that a positive school climate that builds on existing peer and teacher support is associated with lower levels of aggression [40,43]. The first finding is in line with theories of social networks and social support, confirming that higher schoolmate and teacher support during adolescence in school can have a beneficial effect for healthy development [8]. This research shows that this applies not only to emotional, but also to behavioural problems. Our results show that school and teacher support play a crucial role in adolescents with EBP.

We did not find any statistically significant interactions of ACEs and with schoolmate support and with teacher support regarding EBP. Our results showed that schoolmate and teacher support are associated with EBP, but they do not moderate the association between ACE and EBP. This may be explained in several ways. Firstly, through the theory of social support. Even though social support in childhood may have benefits for mental health in adolescents, this is very dependent on the type of support provided (e.g., emotional, instrumental, or informational) [44] and whether the type of support provided is in line with the needs of adolescent who have experienced ACEs. For example, these adolescents may require emotional support from a teacher, even though the teacher only provides more instrumental or informational support [44], or vice versa. Whether the support provided will be effective and impart a buffering influence strongly depends on both an adolescents’ individual preferences for the type and amount of social support and on the actual support provided by teachers and classmates [45]. In order to achieve a buffering effect, these two aspects need to be in accordance, and this is frequently not the case. A second explanation for the missing buffering effect may be that teachers and classmates provide social support to those adolescents with EBP, but that these adolescents do not know how to use this type of help. In this explanation, the ACEs overwhelm these adolescents to such an extent that they are unable to accept help and support from school [46,47]. The third *n* explanation may be that the buffer effect of schoolmate and teacher support in the association between ACE and EBP may not be present for older adolescents with ACEs in the same way as was shown by a previous study among younger adolescents [48]. This later explanation could be biased by the way in which ACEs were questioned (retrospective report of ACEs vs. recent and/or current report of EBPs) and the way in which schoolmate and teachers support were investigated.

A good method by which to build and enhance existing teacher and classmate support is through the help of school psychologists present at school, who undertake activities aimed at improving relationships. Information from teachers, as collected in the Care4Youth qualitative study [49], shows that it is necessary to build a multidisciplinary team at the school, which should consist of school psychologists, social educators, special educators and school social workers. Furthermore, better communication between parents, class teachers [50], and other members of such multidisciplinary teams might add to the prevention of EBP in adolescence [20]. This study did not examine the influence of factors on the community level, but these are expected to be an important source of support with the potential to improve the mental health of adolescents based on theoretical models and already existing knowledge [28,51].

This study has several strengths. The important strengths are its large nationally representative sample and its use of the well-established HBSC methodology. A third strength is that confidentiality as well as privacy were guaranteed by the self-administration of the questionnaires in the absence of teachers, which prevented potential bias through data collection. Moreover, previous research demonstrated the validity of the self-reported measurement of EBP as well as ACE [52,53]. Our study also has some limitations. First, we only used the self-reported data from adolescents, which can be inaccurate and biased due to social desirability and/or recall bias [20]. However, as the questionnaires were anonymously completed and completed without the presence of teachers, this might also have encouraged respondents to provide their answers on rather sensitive topics. Second, the cross-sectional design of this study prevents formulating conclusive statements about the causality of our results. Third, some responses were lacking for some of our variables. We compared the groups with no missing responses with missing responses and found that they differed in that the children in the missing responses group were more often boys and had lower SES. Responses missed were more often for boys and adolescents with lower SES, who may be considered to be at-risk groups. We had expected that having these respondents in the analysis would have led to finding stronger associations, not to weaker ones. Fourth, the SDQ consists of items focused on peer relationship problems, which could overlap to some extent with schoolmate support. However, in this study, the SDQ was used as an overall scale, diminishing the potential of overlap.

## 5. Conclusions

Despite not having found a buffering effect on the association between ACEs and EBP, perceived schoolmate and teacher support was associated with fewer EBPs. As such, school-related support may be of great importance and should be encouraged at schools, as it is expected to have a positive influence on the mental health of adolescents. First, future studies could investigate the moderating effect of schoolmate and teacher support on the relationship between ACE and EBP in younger children, where support could play a more important role than in older adolescents. Second, future research could focus more on the specific type of support provided from school, as these studies may differ. Adolescents may perceive and use support provided in different ways [20]. Previous research shows that teachers mostly provide more informational support but not much emotional support [44], which might be influenced by the skills and competencies of teachers when regarding adolescents with ACE. Through the inclusion of multidisciplinary teams at schools, the provision of support may be divided between teachers and school psychologists. In such cases, teachers can provide more instrumental and/or informational support and school psychologists can provide emotional support while respecting boundaries of competencies of those involved. Finally, the use of school psychologists and multidisciplinary teams in identifying those at risk and intervening early in the school setting could be a way to decrease the likelihood of EBP [20]. However, the effectiveness of such an approach needs to be confirmed. Schoolmate and teacher support decreased the likelihood of EBP among adolescents, but does not buffer the relationship between any previous ACE and EBP.

## Figures and Tables

**Table 1:** Descriptive statistics, Health Behaviour in School-Aged Children study collected in Slovakia 2018.

	Total
	N = 5220
Gender (*n*, %)	
Boys	2542 (48.7)
Age (mean, SD)	13.02 (1.33)
FAS (*n*, %)	
Low	1532 (29.3)
Middle	1578 (30.2)
High	2110 (40.4)
ACE (*n*, %)	
No ACE	799 (15.3)
1–2 ACE	2399 (46.0)
3 or more ACE	2022 (38.7)
Schoolmate support (mean, SD)—range 3–15	10.91 (2.55)
Teacher support (mean, SD)—range 3–15	10.60 (2.89)
Emotional and behavioural problems (*n*, %)	
Normal and borderline	4721 (90.4)
Abnormal	499 (9.6)

HBSC-study—Health Behaviour in School-Aged Children study, N—Number of respondents, FAS—Family affluence, ACE—adverse childhood experiences, SD—Standard Deviation.

**Table 2:** The moderating effect of schoolmate and teacher support on the association between ACE with EBP adjusted for age, gender and FAS (Odds ratios and 95% confidence intervals) (Slovakia 2018, 11–15 years old, N = 5220).

	Emotional and Behavioural Problems	
	Model 1	Model 2
	OR (95% CI)	OR (95% CI)
Schoolmate support		
ACE		
0	Ref.	Ref.
1–2	0.89 (0.64|1.24)	2.02 (0.63|6.46)
3 or more	1.92 (1.40|2.63) ***	3.06 (1.00|9.36) *
Schoolmate support (SS)	0.76 (0.74|0.79) ***	0.81 (0.73|0.89) ***
ACE * Schoolmate support		
1–2 ACE * SS		0.92 (0.82|1.03)
3 ≥ ACE * SS		0.95 (0.85|1.06)
Teacher support		
ACE		
0	Ref.	Ref.
1–2	0.89 (0.64|1.24)	0.56 (0.20|1.55)
3 or more	1.92 (1.40|2.63) ***	0.78 (0.29|2.05)
Teacher support (TS)	0.86 (0.83|0.89) ***	0.80 (0.73|0.88) ***
ACE * Teacher support		
1–2 ACE * TS		1.05 (0.95|1.16)
3 ≥ ACE * TS		1.10 (1.00|1.21)

* *p* < 0.05, *** *p* < 0.001.

## Data Availability

The data presented in this study are available on request from the corresponding author.
